# Size-segregated urban particulate matter: mass closure, chemical composition, and primary and secondary matter content

**DOI:** 10.1007/s11869-015-0359-y

**Published:** 2015-07-15

**Authors:** Wioletta Rogula-Kozłowska

**Affiliations:** Institute of Environmental Engineering, Polish Academy of Sciences, 34 M. Skłodowska-Curie St, 41-819 Zabrze, Poland

**Keywords:** Ambient aerosol, Ultrafine particles, Mass size distribution, Health hazard, PAHs

## Abstract

Forty-nine components of ambient particulate matter (PM) in size-fractionated PM were investigated at an urban background site in Katowice (Silesian Agglomeration in Southern Poland) in the non-heating season of 2012. PM was analyzed for two groups of carbon compounds (organic (OC) and elemental (EC) carbon, Lab OC-EC Aerosol Analyzer), five major water-soluble ions (NH_4_^+^, Cl^−^, SO_4_^2−^, NO_3_^−^, and Na^+^ contents in PM water extracts, ion chromatography), 26 elements (X-ray fluorescence spectrometry), and 16 polycyclic aromatic hydrocarbons (PAHs, gas chromatography). The distributions of the masses of these components among 13 basic PM fractions were determined, and chemical mass closure was checked for each of these fractions separately. The particles having their aerodynamic diameters in the interval 0.03–0.26 μm, the fraction PM_0.03–0.26_, contributed about 13 % to the total PM mass. This PM fraction consisted of primary particles predominantly composed of various inorganic compounds, primary organic compounds, and, in lesser amounts, of elemental carbon, secondary ions, and secondary organic compounds. The second particle fraction, PM_0.26–1.6_, consisted mainly of secondary matter, and its mass contribution to the total PM mass was about 59 %. The third fraction, PM_1.6–40_, was a fraction of coarse particles composed of mineral/soil and organic matter and elemental carbon. It contributed to the PM mass about 28 %. For each of PM_0.03–0.26_, PM_0.26–1.6_, and PM_1.6–40_, the health hazard from its 16 PAH contents was determined by computing toxicity factors. PM_0.26–1.6_ posed the greatest health hazard from the mixture of the 16 PAHs that it contained, PM_1.6–40_ was the next, and the hazard from the PM_0.03–0.26_-bound 16 PAHs was the smallest. The molecular diagnostic ratios computed for these three fractions were specific for coal and wood combustion; some indicated the road traffic effects.

## Introduction

Among all the air pollutants, airborne particulate matter (PM) affects the environment most extensively. PM impacts negatively on climate and human health (Englert 2004; Pope and Dockery 2006; Paasonen et al. 2013; Atkinson et al. 2015).

The PM impact on humans depends on the PM mass and number size distributions. Very small particles of PM (aerodynamic diameters up to 1 μm) are toxic, cytotoxic, and mutagenic; the PM ultrafine fraction (up to 0.1 μm) has the highest oxidative and mutagenic potential (e.g., Massolo et al. 2002; Daher et al. 2014). However, the particle size alone is not decisive in the PM toxicity. Ultrafine PM containing CuO is more harmful to human body cells than micrometric PM, but coarse PM containing TiO_2_ causes genetic damages more often than the finer PM (Karlsson et al. 2009). In fact, several PM properties mutually tangle to produce the synergistic PM toxic potential, and the size distributions and chemical composition seem to be most important.

The chemical composition of PM directly affects the PM volatility, density, reactivity, toxicity, and so on. It accounts for time and space variations in the PM concentrations; it must be taken into account when the PM emissions are to be reduced. Providing basic information on the PM origin, the PM chemistry allows for establishing the source-receptor links.

The problems of chemical composition and identification of the sources of PM and of its particular size fractions have been studied intensely over the last years (e.g., Viana et al. 2008; Putaud et al. 2010; Spindler et al. 2010; Belis et al. 2013; Daher et al. 2014; Kong et al. 2014; Huang et al. 2014; Pokorná et al. 2015). However, neither the chemical composition nor the sources of the finest particles, those with aerodynamic diameters up to 1 μm (PM_1_), are recognized well (Calvo et al. 2013). We know the least about the particles with aerodynamic diameters not greater than 0.1 μm (PM_0.1_) (Sanderson et al. 2014).

The concentrations and chemical composition of PM are very site-dependent; they depend on the local emission sources and the conditions enabling chemical transformations of precursory gaseous compounds. Therefore, the monitoring of the PM concentrations and chemical composition within any area should rely on as dense a network of sampling points as possible. In Central and Eastern Europe, the chemical composition of PM, especially of fine PM (PM_1_, PM_2.5_), is not properly monitored because the adequate sampling points are not numerous (Viana et al. 2008; Putaud et al. 2010; Belis et al. 2013; Calvo et al. 2013; Sanderson et al. 2014).

The present work is a study of 49 PM chemical components in size-fractionated PM at an urban background site in Katowice (Southern Poland). Two groups of carbon compounds, major water-soluble ions, 16 polycyclic aromatic hydrocarbons, and 26 elements (including toxic metals Ni, Cd, Pb, As) were investigated. These chemicals were determined in 13 basic PM size fractions received directly from the impactor: PM_0.03–0.06_, PM_0.06–0.108_, PM_0.108–0.17_, PM_0.17–0.26_, PM_0.26–0.4_, PM_0.4–0.65_, PM_0.65–1_, PM_1–1.6_, PM_1.6–2.5_, PM_2.5–4.4_, PM_4.4–6.8_, PM_6.8–10_, and PM_10–40_ (subscript indexes are the intervals of the particle aerodynamic diameters, μm) and, in some, their superfractions that were defined in the course of the research. Main groups of PM components (mass closure) and contribution of primary and secondary matter and of anthropogenic and natural matter were determined separately for each of the basic fractions and then for some their superfractions. This detailed analysis allowed for the source apportionment of PM emissions in the measuring point neighborhood and provided data on the chemical composition of particular PM fractions, enabling assessment of the health hazard from PM.

## Methods

### Organization of research and research area

**Fig. 1:**
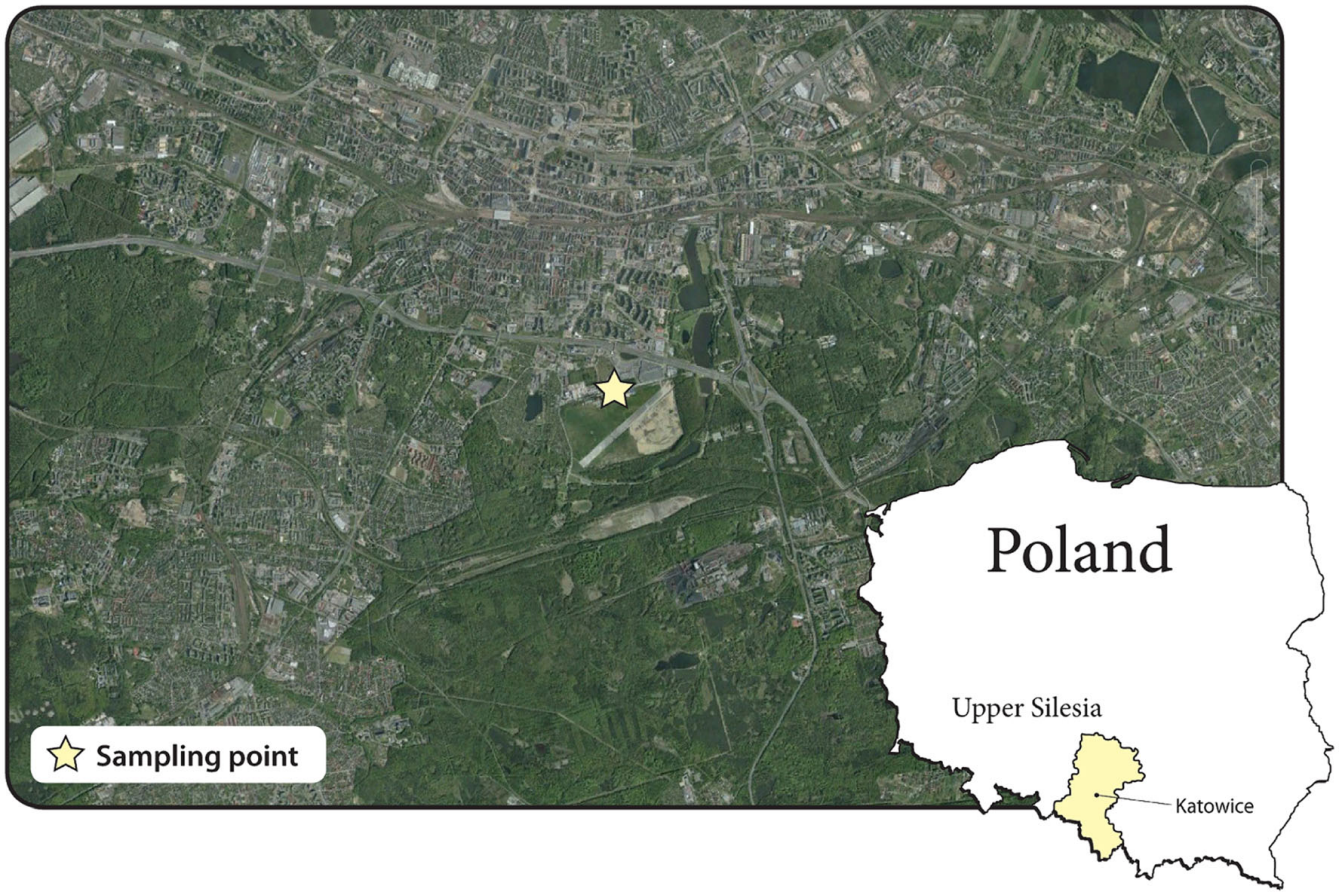
The sampling point location

The area under research was situated within a big living quarter of Katowice (Silesian Agglomeration), beyond the direct effects of industry and road traffic (Fig. 1).

The Silesian Agglomeration lies in the center of Silesia Province, occupies 1230 km^2^, has about 2.1 million population (1691 inhabitants per one square kilometer). It is one of the most urbanized and industrialized regions in Central Europe.

PM was sampled at an urban background sampling point (EC 2008) between the 13 March and the 3 September 2012. Eighteen 13-fold PM samples were taken during the sampling period with the use of a 13-stage DEKATI low-pressure impactor (DLPI, Dekati Ltd.; Kangasala, Finland, flow rate 30 l/min). The particular sample takings lasted from 123 to 173 h; they covered the whole sampling period in about 70 %. There was no sampling in the winter (heating season), because in Silesia, in winter, the PM chemical composition is totally dominated by carbonaceous municipal emissions (mainly elemental carbon) and is entirely different from the PM composition in the rest of the year (Pastuszka et al. 2010; Rogula-Kozłowska and Klejnowski 2013; Rogula-Kozłowska et al. 2014).

Two kinds of substrates were used, both from Whatman (GE Healthcare Bio-Sciences Corp.; Piscataway, NJ, USA). Alternating between the sample takings, QMA quartz fiber filters, ø25 mm, CAT No. 1851-025 (nine samples), and nylon membrane filters, 0.2 μm, ø25 mm, Cat No. 7402-002 (nine samples), were used, the same substrates on all the impactor stages in one sample taking.

Two equal (1.5 cm^2^) fragments were cut out from each exposed quartz filter just before the analysis; PM on one of them was analyzed for organic carbon (OC) and elemental carbon (EC). The remaining fragments were used to make fraction samples, each by putting together all the nine fragments containing the same PM fraction. These 13 fraction samples were analyzed for the following 16 polycyclic aromatic hydrocarbons (PAHs): naphthalene (Na), acenaphthene (Ace), acenaphthylene (Acy), anthracene (An), benzo [a] anthracene (BaA), benzo [a]pyrene (BaP), benzo[b] fluoranthene (BbF), benzo[k] fluoranthene (BkF), benzo[g, h, i] perylene (BghiP), chrysene (Ch), dibenzo[a, h] anthracene (DBA), fluoranthene (Fl), fluorene (F), phenanthrene (Ph), pyrene (Py), and indeno[1,2,3-cd]pyrene (IP).

The PM on the membrane filters was analyzed for the elemental composition (Al, Si, K, Ca, Sc, Ti, V, Cr, Mn, Fe, Co, Ni, Cu, Zn, As, Se, Br, Rb, Sr, Mo, Ag, Cd, Sb, Te, Ba, and Pb). Then, the concentrations of water-soluble ions (Cl^−^, SO_4_^2−^, NO_3_^−^, Na^+^, NH_4_^+^) were determined in the PM water extracts.

### Chemical analyses, QA/QC

The substrates and impactors were prepared for exposure in a laminar chamber. The masses of the PM samples were determined by weighing the substrates before and after the exposure; a MYA 5.3Y.F micro balance (1-μg resolution, RADWAG; Radom, Poland) was used. Before each weighing, the substrates were conditioned for 48 h in the weighing room (relative air humidity 45 ± 5 %, air temperature 20 ± 2 °C). After weighing, the exposed filters were put into petri dishes which were wrapped in light-proof aluminum foil and stored in a freezer at −18 °C till the analysis.

Blank samples were prepared for each of the 13 basic PM fractions. The four-step process of preparing the blanks consisted of (1) taking out 13 filters from their original package and putting them into Petri dishes; (2) 48-h conditioning of the filters in Petri dishes in the weighing room; (3) loading the Dekati impactor with the filters and installing the impactor at the measuring site for 5 days (pump off); (4) removing the filters from the impactor, putting back into Petri dishes, and 48-h conditioning in the weighing room. The process was repeated for both kinds of filters at the beginning and at the end of the measuring period. The blanks were used to determine the detection limits for the analytical methods and the analyte background levels (the amount of the analyte in a blank, μg, determined for each PM basic fraction separately). Neither PM component analytical background was greater than 3.5 % of the component content of the PM sample. The analyte content of a PM sample was received by subtracting its background level value from its amount on the exposed filter.

The OC and EC contents of PM were determined with the use of a Lab OC-EC Aerosol Analyzer (Sunset Laboratories Inc.; Portland, OR, USA) using the EUSAAR protocol. The measurement performance was controlled by systematic calibrating of the analyzer within the range proper for the determined concentrations and by analyzing standards with certified carbon content (RM 8785 and RM 8786, NIST, Gaithersburg, MD, USA) and the blank samples.

The detection limit for total carbon (TC), computed after analyzing the 26 blanks, was 0.52 μg C/cm^2^ (0.43 and 0.09 μg C/cm^2^ for OC and EC, respectively). The standard recovery was from 98 to 122 % of the certified value for OC and from 95 to 116 % for EC (the certified values were taken from the IMPROVE program).

The detailed description of the extraction procedure and the parameters of the chromatographic analysis of PM for PAHs are in given in Rogula-Kozłowska et al. (2013b).

The limits of detection for the 16 PAHs, obtained from the statistical development of the blank results (26 above described quartz fiber filter blanks), were between 6.25 ng (BbF) and 20 ng (Ph).

The method performance was verified by analyzing the NIST SRM1649b reference material and comparing the results with the certified concentrations of the investigated PAHs. The standard recoveries were from 92 % (Ph) to 111 % (Acy).

The elemental composition of PM was determined by means of energy-dispersive X-ray fluorescence (EDXRF). An Epsilon 5 (PANalytical B.V.; Almelo, The Netherlands), calibrated with the use of thin-layer single-element standards (Micromatter; Vancouver, Canada), was used to measure the total concentrations of the elements. To control the performance of the analytical procedure, the NIST SRM2873 samples were measured weekly (except 52 and 39 % recoveries of V and Co, the recoveries were between 85 and 120 % of the certified values) and the X-ray tube and detector drift monitor monthly. The detection limits (from the statistical development of the blank results) were from 0.15 ng/cm^2^ (Se) to 16.8 ng/cm^2^ (Si).

The water extracts of PM were made by ultrasonizing the substrates containing the samples in 25 cm^3^ of de-ionized water for 60 min at the temperature 15 °C and then shaking the extracts for about 12 h (18 °C, 60 r/min). The ion content of the extracts was determined using an ion chromatograph (Metrohm AG; Herisau, Switzerland). The method was validated against the CRM Fluka product nos. 89316 and 89886; the standard recoveries were from 92 % (Na^+^) to 109 % (Cl^−^) of the certified values, and the detection limits were as follows: 10 ng/cm^3^ for NH_4_^+^, 18 ng/cm^3^ for Cl^−^ and SO_4_^2−^, and 27 ng/cm^3^ for NO_3_^−^ and Na^+^.

#### Estimation of secondary matter content of size-segregated PM

The ambient concentrations of fraction-bound secondary organic carbon (OC_sec_), ammonium sulfate ((NH_4_)_2_SO_4_), and ammonium nitrate (NH_4_NO_3_) were determined from the analytically determined amounts of OC, EC, SO_4_^2−^, and NH_4_^+^ in PM.

The mass [OC_sec_]^s*f*^ of the OC_sec_ from the basic fraction *f* in the sample *s* is computed from the equation (Castro et al. 1999):

1 $$ {\left[{\mathrm{OC}}_{\sec}\right]}^{\mathrm{s}f}={{\left[\mathrm{O}\mathrm{C}\right]}^{\mathrm{s}f}}_{\mathrm{A}}-{\left(\frac{{{\left[\mathrm{O}\mathrm{C}\right]}^f}_{\mathrm{A}}}{{{\left[\mathrm{E}\mathrm{C}\right]}^f}_{\mathrm{A}}}\right)}_{\min }.{{\left[\mathrm{E}\mathrm{C}\right]}^{\mathrm{s}f}}_{\mathrm{A}} $$

where

[OC]^s*f*^_A_ is the analytically determined mass of the OC from the fraction *f* in the sample *s*,

[EC]^s*f*^_A_ is the analytically determined mass of the EC from the fraction *f* in the sample *s*,

([OC]^*f*^_A_/[EC]^*f*^_A_)_min_ is the smallest [OC]^s*f*^_A_/[EC]^s*f*^_A_ for all the samples *s* of the fraction *f* (([OC]^*f*^_A_/[EC]^*f*^_A_)_min_ for all the PM fractions are presented in Table 1).

**Table 1:** Minima, maxima, and averages in the sampling period of concentrations of 13 PM fractions, PM-bound OC, EC, five water-soluble ions, 26 elements, and average concentrations of 16 PM-bound PAH (ng/m^3^)

Component	PM_0.03–0.06_		PM_0.06–0.108_		PM_0.108–0.17_		PM_0.17–0.26_		PM_0.26–0.4_		PM_0.4–0.65_		PM_0.65–1_		PM_1–1.6_		PM_1.6–2.5_		PM_2.5–4.4_		PM_4.4–6.8_		PM_6.8–10_		PM_10–40_	
PM	173.46^a^	408.31^b^	186.33	665.10	220.55	1498.44	849.40	3884.98	1327.67	6424.10	1358.28	11,924.88	1013.93	11,474.96	608.36	11,060.25	436.18	3861.50	1201.41	4119.72	574.32	1897.50	187.61	1627.11	187.61	6087.92
	277.25^c^		408.76		734.10		1711.72		2944.81		5120.00		3811.44		2641.93		1656.67		1953.78		1224.73		773.47		1375.40	
OC	68.12	103.20	78.31	167.00	123.15	367.31	292.11	713.62	707.12	1335.39	1159.08	3018.21	741.34	3378.68	475.33	2599.63	322.67	657.59	295.61	714.35	183.48	516.23	129.59	398.89	126.80	385.77
	84.39		125.79		248.19		531.15		1027.35		1706.69		1473.64		1060.49		431.68		495.58		365.72		246.52		227.39	
EC	4.97	11.08	11.11	18.44	15.35	28.62	17.60	80.98	17.52	195.18	51.02	320.23	42.68	160.51	32.68	112.78	36.90	76.19	56.55	117.51	62.43	130.48	47.19	89.30	27.58	82.00
	7.78		14.47		23.03		49.90		76.75		139.34		83.31		59.72		55.32		97.26		85.20		59.03		50.42	
OC/EC^d^	7.34	17.83	7.05	13.34	7.30	16.67	8.81	16.60	6.16	40.35	4.99	28.92	7.50	33.16	6.31	31.22	5.00	17.20	2.52	8.65	2.31	5.76	2.75	5.01	2.54	6.36
	11.84		8.78		10.83		11.61		19.70		16.33		20.35		18.87		8.85		5.44		4.31		4.10		4.75	
OC_sec_^e^	0.00	60.69	0.00	78.79	0.00	206.43	0.00	247.60	0.00	1032.27	0.00	2357.17	0.00	2223.88	0.00	1888.18	0.00	466.46	0.00	420.66	0.00	296.83	0.00	153.64	0.00	177.82
	27.31		23.87		80.03		91.44		554.77		1011.57		848.47		683.75		155.09		250.91		169.11		84.39		99.54	
Na^+^	<DL^f^	11.83	<DL	27.31	<DL	26.11	<DL	20.81	<DL	24.19	<DL	43.36	0.38	26.07	1.23	28.51	0.38	31.35	5.96	46.42	<DL	22.69	<DL	10.68	<DL	33.31
	2.90		5.32		5.72		4.04		6.18		14.77		9.57		11.45		18.05		24.29		8.56		3.33		7.59	
NH_4_^+^	1.00	14.87	6.03	25.73	1.00	49.67	28.58	124.50	66.26	268.73	109.81	619.59	52.32	779.54	12.34	531.36	7.80	179.07	7.44	74.43	3.57	47.79	3.03	19.88	1.00	32.73
	5.10		14.02		24.44		68.30		147.99		309.89		239.82		125.29		40.41		20.93		16.32		9.02		10.61	
Cl^−^	2.20	41.47	2.58	63.91	2.01	62.41	3.35	101.84	4.02	168.92	18.27	276.51	18.65	286.29	7.08	201.88	8.61	75.79	14.06	86.95	10.23	63.94	7.27	58.43	6.70	75.71
	23.33		28.61		28.53		37.41		52.72		88.87		80.34		59.22		39.78		48.80		36.95		32.37		35.42	
NO_3_^−^	2.97	30.37	5.36	46.52	7.65	70.21	15.40	203.17	27.07	361.01	49.64	765.85	33.51	861.40	19.12	568.75	52.32	248.92	85.32	185.94	47.53	111.42	29.34	56.52	24.03	56.97
	20.00		23.90		34.45		72.21		130.49		267.10		270.60		174.74		114.41		125.66		23.90		39.88		39.33	
SO_4_^2−^	17.41	34.45	28.36	62.88	39.71	120.12	74.28	373.63	151.52	576.57	280.07	1036.50	139.18	1078.21	52.04	812.53	23.34	258.07	23.05	149.04	14.92	69.30	12.53	57.75	17.89	57.91
	28.88		42.58		65.20		163.07		289.50		549.49		391.18		228.17		89.77		74.12		47.70		40.92		43.22	
(SO_4_^2−^ + NO_3_^−^) vs. NH_4_^+g^	0.00		0.02		0.09		0.62		0.92		0.97		0.99		0.95		0.95		0.89		0.83		0.74		0.00	
SO_4_^2−^/NH_4_^+h^	0.85	12.00	0.69	2.24	0.50	28.34	0.37	1.42	0.36	1.14	0.32	0.96	0.34	1.08	0.38	1.87	0.54	2.63	0.75	2.75	0.46	3.48	0.36	5.47	0.51	6.71
	4.47		1.33		3.76		0.93		0.79		0.73		0.78		1.12		1.40		1.83		1.71		2.53		2.66	
(NH_4_)_2_SO_4_^i^	0.00		0.00		0.00		102.50	456.91	209.10	795.66	386.50	1430.37	192.06	1487.93	45.29	1121.29	28.61	356.14	27.29	205.67	13.12	94.08	11.14	63.62	0.00	
							201.54		394.94		758.30		535.69		279.80		101.59		69.58		43.20		43.20			
NH_4_NO_3_^j^	0.00		0.00		0.00		0.00	281.34	0.00	665.32	24.22	1618.28	0.00	1815.18	0.00	1099.04	0.00	368.12	0.00	83.93	0.00	99.40	0.00	37.88	0.00	
							60.35		183.26		466.88		421.86		220.05		57.20		9.00		20.38		4.99			
∑_anions_/∑_cations_^k^	0.03	0.73	0.25	0.79	0.29	0.90	0.45	0.84	0.70	1.02	0.79	1.11	0.58	1.04	0.36	1.01	0.33	1.02	0.22	0.79	0.17	0.76	0.07	0.97	0.10	0.85
	0.25		0.48		0.57		0.68		0.84		0.94		0.84		0.63		0.57		0.42		0.40		0.31		0.36	
Al	1.83	2.29	1.44	2.21	1.34	2.29	1.02	2.40	0.16	3.24	<DL	6.37	<DL	5.73	2.26	25.27	6.61	33.55	9.99	38.89	7.18	24.80	3.98	11.87	4.46	16.63
	2.06		1.78		1.85		1.52		1.15		0.91		1.32		8.28		13.91		21.40		15.30		7.91		9.91	
Si	1.38	2.82	1.46	2.81	1.09	2.58	1.11	3.44	1.12	5.05	3.83	16.73	5.28	37.89	20.82	89.46	19.90	103.99	36.34	132.18	25.39	88.79	14.00	44.70	15.40	71.39
	1.97		1.93		1.79		1.95		3.10		10.23		16.01		37.09		46.59		78.28		54.94		28.08		37.95	
K	0.67	1.63	1.46	3.02	1.50	4.10	3.27	10.29	6.21	20.26	14.54	57.01	10.14	40.21	8.06	27.66	5.54	18.32	10.14	22.31	6.06	13.97	2.06	5.83	2.01	23.14
	1.04		2.11		2.59		5.86		11.91		31.28		24.35		18.50		10.44		14.75		9.24		3.89		6.47	
Ca	1.34	2.03	1.42	1.88	1.30	1.88	1.62	2.21	1.97	3.09	3.55	6.87	5.93	12.19	13.30	33.71	15.60	41.51	31.97	115.58	25.30	77.15	14.88	42.43	14.49	449.51
	1.50		1.56		1.58		1.86		2.37		5.22		8.53		20.41		26.36		57.43		44.61		25.99		77.51	
Sc	0.38	0.58	0.37	0.55	0.39	0.54	0.35	0.58	0.28	0.52	0.19	0.47	0.60	1.00	1.34	3.41	1.68	4.46	3.38	12.85	2.67	8.26	1.64	4.67	1.69	54.62
	0.47		0.49		0.47		0.47		0.39		0.38		0.74		2.06		2.77		6.14		4.74		2.95		9.27	
Ti	10.41	17.17	10.71	15.14	11.04	16.18	11.52	16.66	10.13	16.46	9.99	17.55	11.83	16.22	11.70	21.64	13.69	19.96	12.63	22.10	13.28	20.42	11.20	18.26	11.03	20.32
	14.25		13.53		14.08		14.25		13.20		13.11		14.47		15.47		16.71		17.44		15.23		15.38		14.83	
V	1.31	2.14	1.39	1.97	1.38	2.15	1.52	2.09	1.27	2.15	1.47	2.37	1.58	2.30	1.52	2.88	1.91	2.82	1.73	3.35	1.79	2.75	1.56	2.46	1.47	2.76
	1.78		1.71		1.80		1.82		1.72		1.84		1.98		2.13		2.29		2.39		2.05		2.04		1.98	
Cr	0.42	0.89	0.33	0.83	0.54	0.84	0.41	0.83	0.53	0.87	0.54	1.05	0.68	1.04	0.42	1.11	0.67	1.07	0.64	1.19	0.56	0.92	0.58	0.89	0.54	2.32
	0.65		0.59		0.71		0.62		0.68		0.78		0.83		0.84		0.79		0.92		0.77		0.74		1.02	
Mn	2.91	4.90	3.01	4.16	2.93	4.37	3.22	4.64	3.17	4.67	3.75	5.89	4.62	6.46	4.07	7.25	4.20	7.95	3.85	11.93	4.39	8.94	3.26	6.27	3.43	22.19
	3.92		3.73		3.86		4.02		3.91		4.99		5.37		5.65		5.56		6.78		5.44		4.82		6.81	
Fe	0.94	1.57	1.61	2.42	1.59	3.80	3.43	6.27	5.79	10.20	15.84	33.29	19.60	43.82	33.61	119.13	29.22	90.06	49.36	172.98	29.21	84.38	13.74	43.93	12.89	87.74
	1.24		1.90		2.29		4.54		8.11		21.81		30.75		64.09		58.60		98.77		59.35		27.31		37.16	
Co	0.20	0.38	0.16	0.36	0.21	0.34	0.17	0.42	0.09	0.42	0.15	0.38	0.07	0.40	<DL	0.42	<DL	0.50	<DL	0.51	<DL	0.51	<DL	0.39	0.04	0.33
	0.28		0.28		0.27		0.29		0.25		0.26		0.27		0.27		0.28		0.28		0.28		0.24		0.22	
Ni	0.01	0.04	0.03	0.04	0.03	0.05	0.03	0.07	0.05	0.11	0.08	0.19	0.05	0.23	0.05	0.26	0.05	0.12	0.06	0.26	0.05	0.15	0.03	0.09	0.04	2.72
	0.03		0.03		0.04		0.04		0.07		0.13		0.10		0.12		0.08		0.13		0.09		0.06		0.43	
Cu	0.23	0.70	0.30	0.68	0.37	0.71	0.49	0.96	0.71	1.59	1.31	4.24	1.07	5.17	1.36	6.28	0.96	3.66	1.11	3.06	0.68	1.97	0.46	1.10	0.42	2.33
	0.42		0.48		0.51		0.70		1.12		2.64		2.82		2.98		1.72		1.83		1.29		0.71		0.82	
Zn	0.05	0.45	0.27	0.69	0.33	1.25	0.95	4.90	2.40	10.17	6.93	30.81	6.38	35.67	6.95	38.18	3.71	14.57	3.41	18.41	1.48	12.02	0.68	5.15	0.69	30.47
	0.18		0.42		0.54		1.84		4.07		12.82		13.66		14.78		7.13		7.67		4.52		2.08		5.41	
As	0.19	0.46	0.26	0.66	0.19	0.49	0.35	1.12	0.60	1.94	1.40	4.20	1.15	5.84	0.87	8.79	0.35	2.91	0.37	2.76	0.22	1.30	0.15	0.83	0.16	1.12
	0.29		0.39		0.27		0.51		0.86		2.11		2.13		2.56		1.12		1.00		0.55		0.34		0.47	
Se	<DL	<DL	<DL	<DL	<DL	<DL	<DL	<DL	<DL	0.14	0.03	0.42	<DL	0.51	<DL	0.50	<DL	0.03	<DL	0.03	<DL	<DL	<DL	<DL	<DL	<DL
	<DL		<DL		<DL		<DL		0.02		0.19		0.12		0.08		<DL		<DL		<DL		<DL		<DL	
Br	0.32	0.87	0.32	0.97	0.19	0.57	0.40	1.35	0.52	2.09	0.95	4.75	0.43	4.60	0.19	2.47	0.07	0.53	0.03	0.34	0.08	0.23	0.04	0.19	0.03	0.51
	0.54		0.61		0.32		0.61		0.96		2.13		1.44		0.70		0.22		0.16		0.15		0.12		0.14	
Rb	0.01	0.07	0.02	0.09	0.01	0.04	0.01	0.16	0.03	0.30	0.08	0.74	0.05	0.68	0.01	0.34	0.01	0.08	0.04	0.11	0.02	0.08	0.01	0.04	0.01	0.16
	0.03		0.05		0.03		0.05		0.10		0.30		0.20		0.11		0.04		0.06		0.04		0.02		0.04	
Sr	0.20	0.41	0.20	0.44	0.13	0.46	0.16	0.38	0.12	0.47	0.14	0.38	0.13	0.39	0.20	0.61	0.12	0.65	0.23	1.06	0.31	0.72	0.16	0.51	0.20	1.68
	0.29		0.29		0.25		0.22		0.25		0.26		0.26		0.36		0.37		0.61		0.47		0.32		0.53	
Mo	0.01	0.30	0.01	0.31	0.04	0.23	0.01	0.28	0.07	0.27	0.07	0.41	0.03	0.30	<DL	0.30	<DL	0.27	0.03	0.48	0.03	0.21	<DL	0.16	0.03	0.35
	0.10		0.15		0.14		0.18		0.14		0.20		0.16		0.18		0.17		0.24		0.14		0.08		0.20	
Ag	0.20	0.46	0.10	0.46	0.19	0.51	0.13	0.46	0.17	0.44	0.24	0.57	0.19	0.47	0.22	0.34	0.22	0.68	0.18	0.45	0.20	0.48	0.10	0.70	0.20	0.48
	0.31		0.29		0.31		0.27		0.30		0.34		0.31		0.28		0.37		0.33		0.33		0.34		0.33	
Cd	0.22	0.50	0.27	0.52	0.27	0.58	0.27	0.46	0.24	0.69	0.24	1.08	0.28	0.91	0.30	1.99	0.16	0.62	0.22	0.73	0.28	0.51	0.18	0.58	0.33	0.84
	0.37		0.40		0.46		0.35		0.47		0.59		0.57		0.66		0.40		0.43		0.41		0.40		0.44	
Sb	5.68	9.07	5.82	8.11	5.85	8.50	5.89	8.58	5.92	8.81	6.48	10.50	6.74	9.86	6.23	9.15	6.42	9.52	5.49	9.68	5.70	9.64	5.65	9.44	5.20	9.08
	7.35		7.00		7.29		7.67		7.29		8.22		8.10		7.62		7.72		7.40		6.88		7.51		6.91	
Te	0.01	0.46	0.03	0.45	0.10	0.46	0.05	0.46	0.07	0.38	0.05	0.26	0.14	0.43	0.03	0.48	0.01	0.35	0.04	0.61	0.13	0.40	0.01	0.38	0.07	0.35
	0.18		0.17		0.26		0.23		0.20		0.15		0.27		0.22		0.21		0.20		0.24		0.26		0.22	
Ba	1.36	2.33	1.34	2.03	1.25	2.51	1.19	2.21	1.30	1.78	1.44	2.39	1.38	2.57	1.84	4.47	1.64	3.36	1.86	4.73	1.80	3.47	1.39	2.49	1.50	2.53
	1.76		1.65		1.71		1.75		1.55		1.75		1.94		2.59		2.39		3.07		2.49		1.96		1.96	
Pb	0.55	1.39	0.66	1.80	0.39	1.18	0.97	2.93	1.62	5.17	3.51	11.36	2.90	16.50	2.39	26.72	1.15	8.95	0.96	8.48	0.54	3.86	0.15	2.60	0.32	3.24
	0.85		1.05		0.68		1.39		2.34		5.61		6.00		7.58		3.28		2.97		1.59		0.96		1.34	
∑PAH:^l^	0.29		0.25		0.29		0.41		0.58		2.22		2.22		2.11		0.62		1.37		0.62		0.23		0.20	
Na	0.047		0.036		0.043		0.044		0.041		0.050		0.052		0.046		0.029		0.034		0.056		0.027		0.038	
Acy	<DL		<DL		<DL		<DL		<DL		0.034		0.020		0.027		<DL		<DL		<DL		<DL		<DL	
Ace	<DL		<DL		<DL		<DL		<DL		0.013		0.007		<DL		<DL		<DL		<DL		<DL		<DL	
Flu	<DL		<DL		<DL		<DL		<DL		0.009		<DL		<DL		<DL		<DL		<DL		<DL		<DL	
Ph	0.002		<DL		<DL		<DL		<DL		0.048		0.039		0.044		<DL		<DL		<DL		<DL		<DL	
An	0.004		<DL		<DL		<DL		<DL		0.020		0.013		0.024		<DL		<DL		<DL		<DL		<DL	
Fl	0.099		0.076		0.088		0.101		0.109		0.395		0.373		0.329		0.105		0.092		0.168		0.057		0.052	
Py	0.036		0.042		0.028		0.069		0.064		0.332		0.579		0.530		0.092		0.096		0.024		0.019		0.008	
BaA	0.037		0.041		0.056		0.096		0.149		0.403		0.341		0.237		0.258		0.466		0.207		0.068		0.056	
Ch	0.040		0.031		0.035		0.043		0.046		0.337		0.239		0.450		0.023		0.042		0.022		0.016		0.020	
BbF	<DL		<DL		<DL		<DL		0.021		0.095		0.066		0.071		<DL		<DL		<DL		<DL		<DL	
BkF	<DL		<DL		<DL		<DL		0.014		0.072		0.056		0.050		<DL		<DL		<DL		<DL		<DL	
BaP	0.026		0.027		0.036		0.061		0.095		0.282		0.288		0.214		0.110		0.646		0.145		0.045		0.031	
DBA	<DL		<DL		<DL		<DL		0.027		0.040		0.042		0.034		0.008		<DL		<DL		<DL		<DL	
BghiP	<DL		<DL		<DL		<DL		0.012		0.063		0.070		0.043		<DL		<DL		<DL		<DL		<DL	
IP	<DL		<DL		<DL		<DL		0.007		0.034		0.039		0.015		<DL		<DL		<DL		<DL		<DL	

^a^Minimum concentration in March–August 2012

^b^Maximum concentration in March–August 2012

^c^Average concentration in March–August 2012

^d^Proportion of the ambient concentrations of OC and EC

^e^Ambient concentrations of organic carbon from secondary compounds (estimated from Eq. 1)

^f^Concentration lower than the detection limit of the method

^g^Coefficient *R* ^2^ (*p* < 0.05) of the linear correlation between the sum of NO_3_^−^ and SO_4_^2−^ (NO_3_^−^ + SO_4_^2−^; in neq/m^3^) and NH_4_^+−^ (neq/m^3^)

^h^Proportion of the equivalent concentrations of SO_4_^2−^ and NH_4_^+^

^i^Concentration of (NH_4_)_2_SO_4_ (from Eqs. 2 and 5)

^j^Concentration of NH_4_NO_3_ (from Eqs. 3 and 6)

^k^Proportion of the equivalent concentrations of (Na^+^+NH_4_^+^) and (Cl^−^ + NO_3_^−^ + SO_4_^2−^)

^l^Sum of the average ambient concentrations of 16 following PAHs: naphthalene (Na), acenaphthene (Ace), acenaphthylene (Acy), anthracene (An), benzo[a] anthracene (BaA), benzo[a]pyrene (BaP), benzo[b] fluoranthene (BbF), benzo[k] fluoranthene (BkF), benzo[g, h, i] perylene (BghiP), chrysene (Ch), dibenzo[a, h] anthracene (DBA), fluoranthene (Fl), fluorene (F), phenanthrene (Ph), pyrene (Py), and indeno[1,2,3-cd]pyrene (IP)

The masses [(NH_4_)_2_SO_4_]^s*f*^ and [NH_4_NO_3_]^s*f*^ of the (NH_4_)_2_SO_4_ and NH_4_NO_3_ in the basic fraction *f* in the sample *s* are computed from the following formulas (Cheng et al. 2005):

When the proportion of the concentrations of SO_4_^2−^ and NH_4_^+^ (neq/m^3^; Table 1) is less than 1,

2 $$ {\left[{\left({\mathrm{NH}}_4\right)}_2{\mathrm{SO}}_4\right]}^{\mathrm{s}f}=1.38{{\left[{{\mathrm{SO}}_4}^{2-}\right]}^{\mathrm{s}f}}_{\mathrm{A}} $$

3 $$ {\left[{\mathrm{NH}}_4{\mathrm{NO}}_3\right]}^{\mathrm{s}f}=4.44{\left[\mathrm{ex}-{{\mathrm{NH}}_4}^{+}\right]}^{\mathrm{s}f} $$

4 $$ {\left[\mathrm{ex}-{{\mathrm{NH}}_4}^{+}\right]}^{\mathrm{s}f}={{\left[{{\mathrm{NH}}_4}^{+}\right]}^{\mathrm{s}f}}_{\mathrm{A}}-0.27{\left[{\left({\mathrm{NH}}_4\right)}_2{\mathrm{SO}}_4\right]}^{\mathrm{s}f} $$

where

[SO_4_^2−^]^s*f*^_A_ is the analytically determined mass of SO_4_^2−^ from the fraction *f* in the sample *s*,

[NH_4_^+^]^s*f*^_A_ is the analytically determined mass of NH_4_^+^ from the fraction *f* in the sample *s*,

[ex-NH_4_^+^]^s*f*^ amount (mass) of NH_4_^+^ from the fraction *f* left after the reaction with SO_4_^2−^ (excessive NH_4_^+^) in the sample *s*,

When the proportion of the concentrations of SO_4_^2−^ and NH_4_^+^ (neq/m^3^; Table 1) is not less than 1,

5 $$ {\left[{\left({\mathrm{NH}}_4\right)}_2{\mathrm{SO}}_4\right]}^{\mathrm{s}f}=3.67\ {{\left[{{\mathrm{NH}}_4}^{+}\right]}^{\mathrm{s}f}}_{\mathrm{A}} $$

6 $$ {\left[{\mathrm{NH}}_4{\mathrm{NO}}_3\right]}^{\mathrm{s}f}=0 $$

## Results and discussion

### Concentration and mass size distribution of PM and PM components

The minima, maxima, and averages in the measuring period of ambient concentrations for each substance determined analytically in each basic PM fraction, except for the 16 PAHs whose extreme concentrations were not measured, are presented in Table 1; together with the values of some parameters employed in computing, these values for the ambient concentrations of OC_sec_, (NH_4_)_2_SO_4_, and NH_4_NO_3_ are also presented in Table 1.

In Katowice, the core PM mass was due to PM_0.26–1.6_ (Table 1). The measuring period average PM_0.26–1.6_ concentration was 14.5 μg/m^3^, about 60 % of the PM_10_ concentration. The remaining 40 % was divided between PM_0.03–0.26_ and PM_1.6–10_ in the proportion of 1:2. The masses of the main PM components were also concentrated in PM_0.26–1.6_. By mass, about 50 % of Cl^−^, 60 % of NO_3_^−^, 70 % of OC and SO_4_^2−^, and 80 % of Na^+^ were in PM_0.26–1.6_, from 11 % (Na^+^) to 21 % (Cl^−^) in PM_0.03–0.26_, and from 10 % (Na^+^) to 40 % (EC) in PM_1.6–10_.

**Fig. 2:**
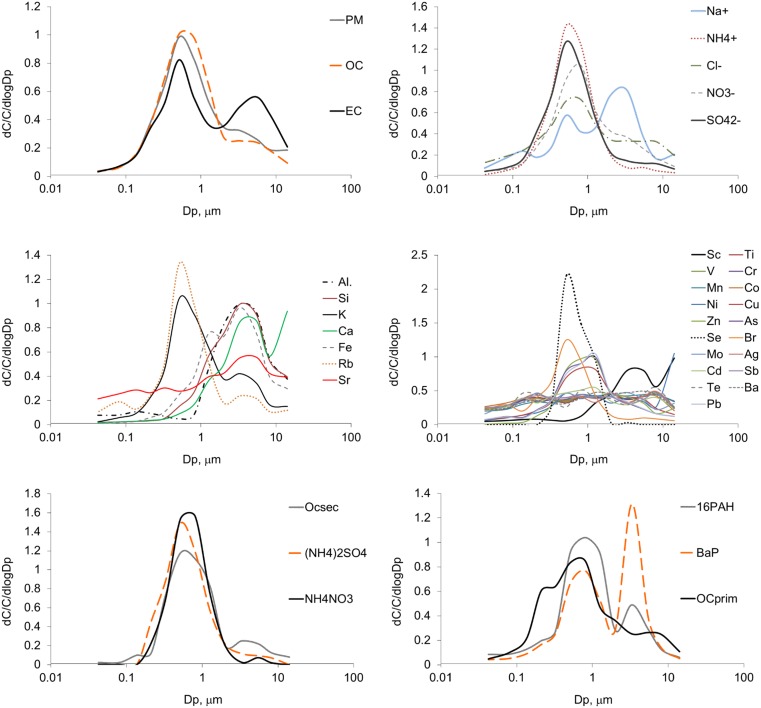
Mass size distribution of PM and its components (*dC* PM or PM fraction-bound component concentration, *C* average concentration of PM or PM-bound component, *Dp* particle aerodynamic diameter)

The density function of the PM mass distribution with respect to particle size has its absolute (greatest local) maximum at the point (the greatest frequency mode, main mode) between 0.4 and 0.65 μm (Fig. 2). The lack of a mode within the greatest diameters indicates the relatively small share of natural mineral components in PM. Nucleation was not a significant PM source during the measuring period either (Friedlander 1970, 1971; Seinfeld and Pandis 2006). Nevertheless, for both PM_6.8–40_ and PM_0.108–0.4_, the proportion of the maximum to minimum concentrations in the measuring period is high, so, the activity of natural mineral matter and nucleation as PM sources varied quite widely not affecting visibly the PM mass size distribution. Similar results were obtained for summer in Zabrze, about 15 km east of Katowice (Klejnowski et al. 2012).

The PM-bound OC, EC, NH_4_^+^, SO_4_^2−^, Cl^−^, and NO_3_^−^ mass size distributions have their main modes in the interval 0.4–1 μm, like the distribution of PM (Fig. 2). The distribution of EC is bimodal (the second mode for EC is in 4.4–6.8 μm); the rest are unimodal. The Na^+^ distribution is trimodal; its main mode is in the interval 2.5–4.4 μm. The two other modes are in 0.108–0.17 μm and 0.65–1 μm. While the presence of the mode for Na^+^ in 2.5–4.4 μm is accounted for by the road dust or soil contamination with NaCl (Pant and Harrison 2013; Li et al. 2014), the mode for EC within such great diameters is not typical; it should rather be expected within very small diameters (ultrafine particles), as the traffic emissions are dominant in summer (Maricq 2007; Alves et al. 2015).

Most probably, the distance caused the traffic EC to reach the sampling point as big particles of soot; EC in coarse particles may be attributed to traffic-induced non-exhaust emissions (Amato et al. 2014). Moreover, the effect of emissions from household coal combustion, although weaker than in winter, cannot be totally excluded. Household ovens, active in the region in the whole year, release big particles of soot from incomplete hard coal combustion. These particles adsorb great amounts of light organic compounds on their surface, contributing to PM-bound OC; the mass distributions of PM-bound secondary organic carbon (OC_sec_) and of primary organic carbon (OC_prim_) have the modes in the interval of big particle diameters.

Except for Na^+^, PM-bound water-soluble ions have unimodal mass size distributions (Fig. 2). The ratios *Σ*_anions_/*Σ*_cations_ of the total anion concentration $\left({\varSigma}_{\mathrm{anions}},\ \frac{\mathrm{neq}}{{\mathrm{m}}^3}\right)$ to the total cation concentration $\left({\varSigma}_{\mathrm{anions}},\ \frac{\mathrm{neq}}{{\mathrm{m}}^3}\right)$, both minimum and average, are smaller than 1 for all the basic fractions (Table 1). Because *Σ*_anions_ and *Σ*_cations_ are highly linearly correlated (*R*^2^ = 0.98, *p* < 0.05), SO_4_^2−^, NO_3_^−^, and Cl^−^ were most probably in compounds with Na^+^ and NH_4_^+^ in majority of the fractions (Seinfeld and Pandis 2006; Kong et al. 2014). The coarse particles (to a lesser extent ultrafine particles too) contained probably NaCl and NaNO_3_ (modes of the Cl^−^ and NO_3_^−^ distributions were shifted toward big particle diameters relative to other ion distribution modes); Na_2_SO_4_, NH_4_NO_3_, (NH_4_)_2_SO_4_, and NH_4_Cl were mainly in fine PM (but in coarse particles too). Obviously, in PM, the three determined water-soluble ions, SO_4_^2−^, NO_3_^−^, and Cl^−^, can also occur bound to chemicals other than water-soluble ions, different in different fractions.

The majority of the mass size distributions of PM-bound elements are bi- or tri-modal (Fig. 2). The distributions of Se, Br, Pb, Cu, Zn, As, and K, the elements related to solid (including coal and biomass) and liquid fuel combustion (Chow 1995; Kumar et al. 2013; Sanderson et al. 2014; Zhang et al. 2014), have very distinctive (of high frequency) modes in the interval 0.4–0.65 μm. Some have the modes in the interval of smaller diameters, like those for Mn, Fe, Co, Cd, Ni, V, and Mo, the elements released probably by traffic (Geller et al. 2006; Sanderson et al. 2014) and industry (Chow 1995; Kumar et al. 2013) as oxides which aggregate to form the particle nucleation mode in the air. Majority of the element distributions have also the modes in the interval of greater diameters, proving their partially non-exhaust origin (Adachi and Tainosho 2004; Wahlin et al. 2006; Pant and Harrison 2013). The elements such as Al, Si, and Ca, whose distributions have the modes in the interval of great diameters, came probably from soil/mineral matter resuspension.

### Chemical mass closure of size-resolved PM

The analytically determined PM components can occur in PM as chemical elements, in chemical compounds with other PM components, or, not numerous, they are chemical compounds themselves. Quite naturally, they fall into five categories according to their origin and the analytical methods used to find their masses in the PM samples: organic carbon (OC) from PM-bound organic compounds; elemental carbon (EC) from incomplete combustion; secondary ions SO_4_^2−^, NO_3_^−^, and NH_4_^+^; crustal elements, and anthropogenic (trace) elements. Their masses in PM samples, being the sums of the analytically received masses of their members, account for the greater part of the PM mass (Table 1), but there is still some deficient mass, the mass of all the substances from beyond these categories, which can be lowered by taking into account the origin of the analytically determined elemental PM components and which is also significant in the chemical content of PM.

Organic carbon (OC) comes from PM-bound organic compounds that can be divided into secondary organic matter (SOM) and primary organic matter (POM). Their masses, [SOM] and [POM], in the sampled PM are computed from the mass [OC]_A_ of the analytically determined OC and the mass [OC_sec_] of secondary organic carbon (OC_sec_, namely [SOM] = 1.6 [OC_sec_] and [POM] = 1.2 ([OC]_A_-[OC_sec_]). A lower conversion factor is taken for the [POM] than for the [SOM] calculation (Turpin and Lim 2001).

For all the basic sub-fractions of PM_0.17–10_, the masses [NH_4_^+^] and [NO_3_^−^] + [SO_4_^2−^] are linearly correlated (Table 1); therefore, in these fractions, some amounts of NH_4_^+^, NO_3_^−^, and SO_4_^2−^ occur probably as (NH_4_)_2_SO_4_ and NH_4_NO_3_. For these basic fractions, the secondary inorganic matter (SIM) is assumed to consist of (NH_4_)_2_SO_4_ and NH_4_NO_3_, and [SIM] = [(NH_4_)_2_SO_4_] + [NH_4_NO_3_]. For the fractions PM_0.03–0.06_, PM_0.06–0.108_, PM_0.108–0.17_, and PM_10–40_, for which the masses [NH_4_^+^] and [NO_3_^−^] + [SO_4_^2−^] are not correlated and, in which, consequently, NH_4_^+^, NO_3_^−^, and SO_4_^2^ were assumed to occur neither as (NH_4_)_2_SO_4_ nor as NH_4_NO_3_, SIM is assumed to be empty and [SIM] = 0.

Finally, mineral matter (MM) and anthropogenic trace matter (ATM) are built of chemical compounds of the PM-bound elements from Table 1: MM consists of the compounds of the crustal elements, and ATM consists of the compounds of the anthropogenic elements.

The groups of crustal and trace elements are defined by dividing all the determined elements into two categories using the enrichment factors (EF; Rogula-Kozłowska et al. 2014) and analyzing their mass size distributions (Fig. 2). For all the basic fractions, Si, Al, and Ca have low EFs, not greater than 16 (Table 2). They accumulated in coarse particles, and their mass size distributions are unimodal with the mode in the interval of the particle diameters greater than 2.5 μm. This and their EFs suggested their crustal origin for all the basic fractions. K, Fe, Rb, and Sr in coarse PM have the EFs low, decreasing with growing particle diameter. Their distributions are bimodal, with one mode in the diameters greater than 2.5 μm, like the distributions of Si, Al, and Ca, the second one in the diameters less than 1 μm. The second mode indicates the presence of an anthropogenic fine particle population (accumulation mode) containing K, Fe, Rb, and Sr. Thus, PM_1–40_-bound K, Fe, Rb, and Sr are crustal, and PM_0.03–1_-bound K, Fe, Rb, and Sr are anthropogenic. The rest of the elements, Cl^−^ and Na^+^, are anthropogenic in each of the basic fractions. They are assumed to be anthropogenic because they have high EFs and the main modes of their distributions are in the particle diameters less than 1 μm. The distributions of Cu, Cr, Mn, Zn, Mo, Cd, Ba, Sb, and Pb have also the modes within the interval of greater diameters, but their anthropogenicity is decided by their high EFs.

**Table 2:** Enrichment factors (EF) for the elements determined in the particular PM fractions (averages in the measuring period)

	PM_0.03–0.06_	PM_0.06–0.108_	PM_0.108–0.17_	PM_0.17–0.26_	PM_0.26–0.4_	PM_0.4–0.65_	PM_0.65–1_	PM_1–1.6_	PM_1.6–2.5_	PM_2.5–4.4_	PM_4.4–6.8_	PM_6.8–10_	PM_10–40_
Al	4	4	4	3	1	0	0	1	1	1	1	1	1
Si	1	1	1	1	1	1	1	1	1	1	1	1	1
K	6	12	15	32	41	32	16	5	2	2	2	1	2
Ca	8	8	9	10	8	5	5	6	6	8	8	10	16
Sc	10,343	11,007	11,384	10,449	5454	1610	2004	2408	2578	3401	3740	4555	10,590
Ti	704	683	766	711	415	125	88	41	35	22	27	53	38
V	5174	5073	5758	5344	3177	1030	708	329	281	175	214	416	299
Cr	2861	2651	3439	2757	1902	661	450	196	147	102	122	229	233
Mn	1146	1113	1242	1187	726	281	193	88	69	50	57	99	103
Fe	6	10	13	23	26	21	19	17	12	12	11	10	10
Co	3718	3796	3946	3891	2110	665	441	190	157	94	133	224	152
Ni	248	254	365	335	368	207	102	53	28	27	27	35	185
Cu	4525	5278	6047	7618	7667	5477	3738	1705	783	496	498	537	459
Zn	533	1270	1761	5507	7662	7314	4980	2326	893	572	480	432	832
As	22,337	30,662	22,888	39,686	42,096	31,297	20,188	10,473	3648	1938	1519	1837	1879
Se	–	–	–	–	23,590	67,909	27,406	7887	–	–	–	–	–
Br	51,992	59,949	33,908	59,334	58,738	39,492	17,060	3580	896	388	518	811	700
Rb	42	71	46	71	89	81	34	8	2	2	2	2	3
Sr	141	144	134	108	77	24	16	9	8	7	8	11	13
Mo	11,004	16,848	16,954	20,010	9790	4238	2166	1052	791	665	552	618	1142
Ag	868,286	829,102	955,600	764,006	533,982	183,388	106,841	41,655	43,820	23,261	33,143	66,811	47,981
Cd	558,812	616,641	764,601	534,027	451,093	171,596	105,929	52,944	25,544	16,344	22,204	42,383	34,496
Sb	3,652,494	3,550,660	3,986,969	3,850,606	2,302,153	786,619	495,293	201,125	162,216	92,544	122,594	261,825	178,252
Te	–	–	–	–	–	–	–	–	–	–	–	–	–
Ba	406	388	434	408	227	78	55	32	23	18	21	32	23
Pb	7703	9712	6782	12,725	13,475	9790	6690	3648	1257	677	517	610	630

The crustal elements occur mainly in oxides and carbonates in the Earth’s crust and soil (Seinfeld and Pandis 2006). The most common such compounds are SiO_2_, Al_2_O_3_, Fe_2_O_3_, CaO, K_2_O SrCO_3_, and CaCO_3_. Rubidium occurs mainly as an ingredient of minerals, where it is never a chief constituent. It is hard to decide in which compounds it occurs in Katowice. So, instead of Rb compounds only, Rb is included into MM. It is also assumed that one half of the analytically determined mass [Ca]_A_ of Ca comes from CaO and the second half from CaCO_3_.

Concluding, for PM_1–40_, [MM] = [SiO_2_] + [Al_2_O_3_] + [Fe_2_O_3_] + [CaO] + [K_2_O] + [CaCO_3_] + [SrCO_3_] + [Rb], and for PM_0.03–1_, [MM] = [SiO_2_] + [Al_2_O_3_] + [CaO] + [CaCO_3_].

The anthropogenic elements in PM come mainly from oxides, sulfides, sulfates, nitrates, chlorates, and fluorides (Chow 1995; Kyotani and Iwatsuki 2002). Sulfides, sulfates, nitrates, chlorates, and fluorides are soluble in water, and oxides are assumed not to be (Rogula-Kozłowska et al. 2013a). ATM comprises SO_4_^2−^, NO_3_^−^, and NH_4_^+^ (i.e., secondary ions, SI; [SI] = [NH_4_^+^] + [NO_3_^−^] + [SO_4_^2−^]), all the anthropogenic elements, and, except for Cl^−^, Na^+^, Se, Br, Sr, Sc, Co, Ag, Rb, Mo, and Te, their oxides. The mass [ATM] of ATM is the sum of the masses of all the substances from these three ATM subcategories.

The part of [SI] in [ATM] is the mass of those ions that were not bound in compounds in SIM; i.e., the mass contribution of SI to [ATM] is equal to [SI]-[SIM].

For three fractions, PM_0.26–0.4_, PM_0.4–0.65_, and PM_0.65–1_, [SI]-[SIM] is negative. It is due to the overestimation of the masses of PM-bound NH_4_(SO_4_)_2_ and HN_4_NO_3_ in the Eqs. 2, 3, 4, 5, and 6, where these compounds are assumed to occur in each PM fraction and that ambient SO_4_^2−^ is entirely, and prior to NO_3_^−^, neutralized by NH_4_^+^. However, only when the molar ratio NH_4_^+^/SO_4_^2−^is not less than 2, the ambient H_2_SO_4_ can be totally neutralized (Seinfeld and Pandis 2006), and PM_2.5_ can be acidic even when this ratio is greater than 2 (SO_4_^2−^/NH_4_^+^ ≤0.5; Pathak et al. 2009; Huang et al. 2011). Therefore, some part of ambient SO_4_^2−^ might not have reacted with NH_3_ and occurred as H_2_SO_4_. The proportions of the concentrations of total anions to total cations (equivalent ion balance, Σ_anions_/Σ_cations_, Table 1), relatively high for these three fractions (still less than 1, but higher than for other fractions), confirm the possibility of the H_2_SO_4_ presence in PM. Another cause of [SI]-[SIM] being negative may be the loss of semi-volatile HN_4_NO_3_ from samples during sampling (almost a whole-week exposure of a filter in summer), but such artifacts are significant only in PM samples very rich in ammonium (Pathak et al. 2009).

Because the absolute values of the negative [SI]-[SIM] do not exceed 1.5 % of the PM mass for any of PM_0.26–0.4_, PM_0.4–0.65_, and PM_0.65–1_, 0 was substituted for the negative [SI]-[SIM] in the formulas for calculation of [ATM].

The mass of the oxides of an element in PM is assumed to be the mass of its most common in PM oxide and is stoichiometrically computed from the mass of this element.

In general, more than 70 % of the mass of each of K, Ti, Zn, Ba, and Pb from a PM sample passes into water (Rogula-Kozłowska et al. 2013a), i.e., is water-soluble. In this paper, only 25 % of their analytically determined in PM masses are assumed to come from insoluble oxides, and such a percentage of their mass is taken to compute the K_2_O, TiO_2_, ZnO, BaO, and PbO masses in ATM. The solubility of each of Cr, Mn, and Sb is between 30 and 50 %, and 50 % of each of their analytical mass in PM is used to calculate the Cr_2_O_3_, MnO, and Sb_2_O_3_ masses in ATM. The solubility of Fe and Cu is less than 30 %; the masses of the insoluble PM-bound Fe_2_O_3_ and CuO in ATM are computed from 75 % of the analytically determined masses of Fe and Cu, respectively. Similarly, the solubility of each of V, Ni, As, and Cd, which had not been considered earlier in (Rogula-Kozłowska et al. 2013a), is assumed to be 25 %, and 75 % of the analytically determined masses of these elements are taken to compute the masses of V_2_O_5_, NiO, As_2_O_5_, and CdO in ATM. Consequently, 75 % of the analytically determined masses of K, Ti, Zn, Ba, and Pb; 50 % of the masses of Cr, Mn, and Sb; and 25 % of the masses of Fe, Cu, V, Ni, As, and Cd are included in [ATM]. They contribute to [ATM] as the masses of these parts of the elements that are in compounds with PM-bound sulfides, sulfates, nitrates, chlorates, etc.

The analytically determined masses of PM-bound Cl^−^, Na^+^, Se, Br, Sr, Sc, Co, Ag, Rb, Mo, and Te are included into [ATM] in total—the mass of Cl^−^ because Cl^−^ does not occur in oxides, the mass of Na^+^ because 100 % of Na is soluble (Rogula-Kozłowska et al. 2013a), and the masses of the rest arbitrarily, because either they do not occur in oxides or their compounds occurring in PM are hard to determine.

Concluding, in PM_1–40_, [ATM] = [TiO_2_] + [ZnO] + [BaO] + [PbO] + [Cr_2_O_3_] + [MnO] + [Sb_2_O_3_] + [CuO] + [V_2_O_5_] + [NiO] + [As_2_O_5_] + [CdO] + 0.75([Ti] + [Zn] + [Ba] + [Pb]) + 0.5([Cr] + [Mn] + [Sb]) + 0.25([Cu] + [V] + [Ni] + [As] + [Cd]) + [Cl^−^] + [Na^+^] + [Se] + [Br] + [Sc] + [Co] + [Ag] + [Mo] + [Te] + ([SI]-[SIM])

And in PM_0.03–1_, [ATM] = [K_2_O] + [TiO_2_] + [ZnO] + [BaO] + [PbO] + [Cr_2_O_3_] + [MnO] + [Sb_2_O_3_] + [Fe_2_O_3_] + [CuO] + [V_2_O_5_] + [NiO] + [As_2_O_5_] + [CdO] + 0.75([K] + [Ti] + [Zn] + [Ba] + [Pb]) + 0.5([Cr] + [Mn] + [Sb]) + 0.25([Fe] + [Cu] + [V] + [Ni] + [As] + [Cd]) + [Cl^−^] + [Na^+^] + [Se] + [Br] + [Sr] + [Sc] + [Co] + [Ag] + [Rb] + [Mo] + [Te] + ([SI]-[SIM]).

For each PM basic fraction, the mass [UM] of the unidentified matter UM is as follows: [UM] = [PM]-([SOM] + [POM] + [EC] + [SIM] + [MM] + [ATM]).

**Fig. 3:**
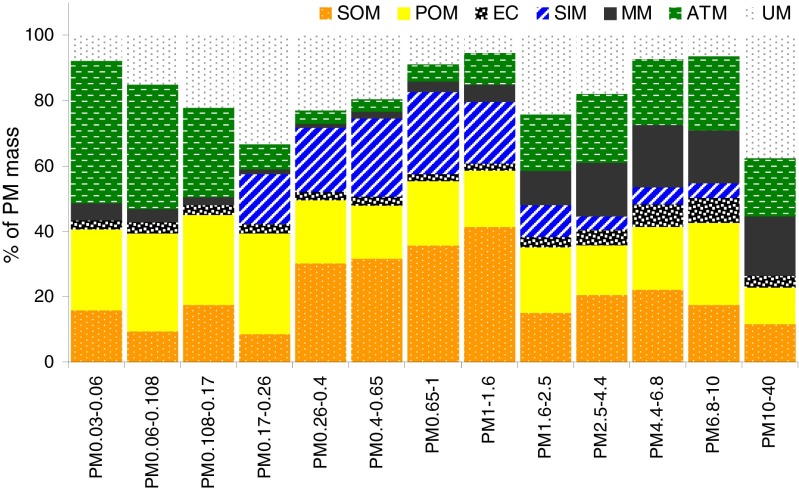
Averaged in the measuring period mass contributions to PM of secondary organic matter (SOM), primary organic matter (POM), elemental carbon (EC), secondary inorganic matter (SIM), mineral matter (MM), anthropogenic trace matter (ATM), and unidentified matter (UM)

The calculations of the masses of the compounds in MM, ATM, and in other categories, too, are somewhat speculative. The assumptions, taken from the cited papers, are simplifications and are neither always nor everywhere true. Moreover, because of alternate use of quartz and membrane substrates, the chemical constituents whose masses were used in the computations were sampled at different times. Thus, Fig. 3 presents only a very rough estimate of the chemical composition of the 13 basic PM fraction in Katowice.

The UM mass share in PM_10–40_, 37.5 %, is the greatest among all the UM shares in the basic fractions (Fig. 3). Most probably, UM consists of water, organic compounds, and nitrates that evaporate during handling of the PM samples and of unidentified compounds (Chow 1995; Tsyro 2005; Seinfeld and Pandis 2006). The inaccuracies in identification of compounds in OM, MM, SIM, and ATM also affect UM. The MM mass content of PM_10–40_ is higher than that of other basic fractions; the ATM content of it is also high, so PM_10–40_ is supposed to be hygroscopic (containing hygroscopic Al_2_O_3_, CaCl_2_, NaCl, etc.) and to contain some unidentified PM-bound water. Also, UM shares in PM_0.17–0.26_ and PM_1.6–2.5_ are significant. The former fraction contains a great share of SIM whose mass could be underestimated in the stoichiometric computations; the latter contains much water, like PM_10–40_, and probably also underestimated amount of OM.

**Fig. 4:**
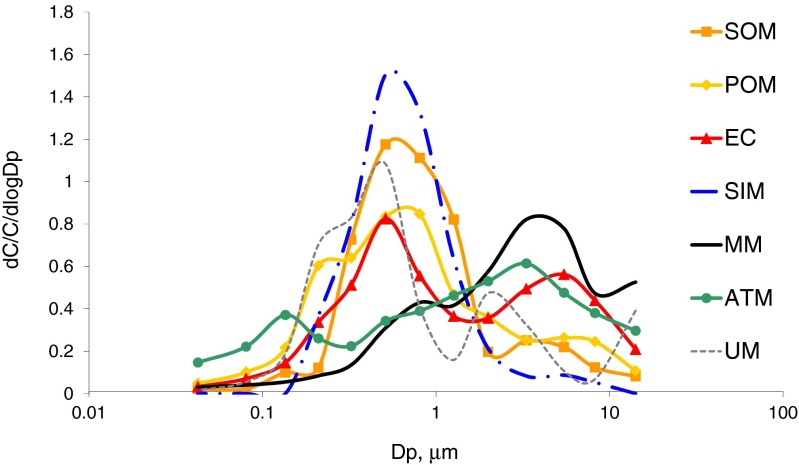
Mass size distributions of secondary organic matter (SOM), primary organic matter (POM), elemental carbon (EC), secondary inorganic matter (SIM), mineral matter (MM), anthropogenic trace matter (ATM), and unidentified matter (UM)

ATM contributes to PM_0.03–0.26_ mass much, about 29 % in average (mode in 0.17—0.26 μm, Fig. 4), to PM_0.03–0.06_ even 44 %. Besides, POM contributes to PM_0.03–0.26_ mass 28 %, EC—3.1 %, and MM—3.2 %, so primary matter makes 64 % of PM_0.03–0.26_. Secondary matter (SOM and SIM) is about 16.6 % in the PM_0.03–0.26_ mass.

The composition of PM_0.26–1.6_ is entirely different from that of PM_0.03–0.26_. SOM and SIM prevail in PM_0.26–1.6._ The mass size distributions of SOM, SIM, EC, and POM have modes in 0.4–0.65 μm (Fig. 4). The average secondary matter mass content (SOM and SIM) in PM_0.26–1.6_ is 56.6 %, and POM, EC, MM, and ATM together are 29.2 %.

The EC mass content of PM_1.6–40_ is about 5.3 % and is much greater than those of PM_0.03–0.26_ and PM_0.26–1.6_; MM and ATM are 16.1 and 19.7 % in PM_1.6–40_. So, in PM_1.6–40_, primary matter is 59.3 % and secondary matter (SOM and SIM) is no more than 22 %. All the group mass size distributions have modes within the interval of great particle diameters.

It has appeared that the PM at the Katowice sampling point consists of three quite sharply differing fractions: PM_0.03–0.26_, PM_0.26–1.6_, and PM_1.6–40_.

PM_0.03–0.26_ is composed of primary metal oxides and salts, primary organic compounds, and, in lesser amounts, of EC and secondary compounds. This PM fraction comes most probably from road traffic and industry (combustion of fossil fuels an biomass in heating and power plants, iron and steel industry, metallurgy) (Geller et al. 2006; Maricq 2007; Sanderson et al. 2014; Kumar et al. 2013), and by mass, it is about 13 % of the total PM.

PM_0.26–1.6_, mainly secondary, contributes to total PM about 59 %. It contains SOM and SIM from transformations of precursory gaseous compounds (from road traffic, solid fuel combustion, industry, etc.). Its ambient concentrations depend strongly on and are very sensitive to the variations of atmospheric conditions (insolation, precipitation, air pressure and temperature, EC concentration, etc.) and ambient concentrations of oxidants (such as ozone) (Seinfeld and Pandis 2006; Pathak et al. 2009; Huang et al. 2011). Even the tendency of their variations is hard to forecast because the factors upon which they depend are interrelated. Nevertheless, in Southern Poland, the secondary matter formation seems to be equally effective relative to the PM concentrations in heating and non-heating periods: in both these periods of 2009 in Zabrze, a city about 15 km east of Katowice, secondary matter was about 50 % of PM_1_ (Rogula-Kozłowska and Klejnowski 2013), and the present study proves it to be 48 % in Katowice in the non-heating period of 2012.

PM_1.6–40_, about 28 % of the PM mass, contains mainly primary coarse particles of mineral/soil and road dust (various salts, hygroscopic aluminosilicates), soot (EC), particles from construction sites, etc. The PM_1.6–40_ concentrations are probably directly proportional to the rates of these particle resuspensions; therefore, they depend on wind, erosion, and corrosion. These big particles can contain volatile and semi-volatile compounds from SOM and SIM on their surfaces. Most probably, the majority of the PM_1.6–40_-bound compounds from SOM and SIM are adsorbed on big particles of soot (EC) agglomerated during transport or released by inefficient household ovens.

### PAHs in size-segregated PM

**Fig. 5:**
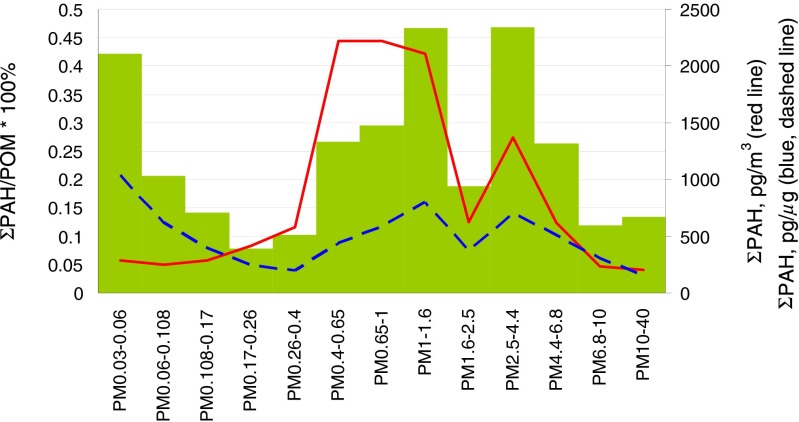
Ambient concentrations of 16 PAH (ΣPAH, pg/m^3^; *red line*) and their share in PM mass (pg/μg; *blue*, *dashed line*) and in POM (%, *bars*)

PM-bound PAHs are in POM; some, in favorable conditions, are precursory to SOM (Zhang and Ying 2012). In Katowice, the 16 determined PAHs (ΣPAH) were no more than 0.1 % of the POM mass in each PM_0.17–0.4_ basic sub-fraction and slightly more than 0.1 % in each basic sub-fraction of PM_6.8–40_ (Fig. 5). In the sub-fractions of PM_0.4–6.8_, they were 0.2–0.47 % and in PM_0.03–0.06_—0.42 %. Among all the 13 basic fraction-bound ΣPAHs, the ambient concentrations of the PM_0.4–1.6_- and PM_2.5–4.4_-bound ΣPAH were the highest and those of the PM_0.03–0.26_-, PM_1.6–2.5_-, and PM_4.4–40_-bound—the lowest (Fig. 5). So, both the ambient concentrations and the shares in POM of ΣPAH from three out of all four basic sub-fractions of the PM_0.26–1.6_ (except PM_0.26–0.4_) were relatively high.

The ambient concentrations of ΣPAH from the basic sub-fractions of PM_0.03–0.26_ and PM_1.6–40_ were low, but the ΣPAH contents of POM and of PM were high in some of them. In the interval 0.03–0.26 μm, ΣPAH contents of POM and of PM in basic fractions decrease and the ΣPAH ambient concentration grows with growing particle diameter. In 0.26–1.6 μm, the concentrations of ΣPAH and ΣPAH content of both POM and PM grow with growing particle diameter; in 1.6–40 μm, they rather decrease. Despite possible artifacts from sampling (related to semi-volatile PAHs), the behavior of PM-bound ΣPAH partitions again PM into PM_0.03–0.26_, PM_0.26–1.6_, and PM_1.6–40_ at the site in Katowice.

Like POM, ΣPAH has a bimodal mass size distribution; the main mode is in the interval 0.65–1 μm of the diameters of the particles contained in PM_0.26–1.6_ and rich in EC; the second one is in the interval 1.6–40 μm of the diameters of big particles rich in EC and MM (Fig. 4).

Despite relatively low mass contribution of PAHs to POM, and very low to PM, PM-bound PAHs can be characteristic of a PM source and can be used to trace the origin of PM (Ravindra et al. 2008; Tobiszewski and Namieśnik 2012). They may be used as markers or molecular diagnostic ratios which can be computed based on the PAH concentrations. The markers of a source are the PAHs that are characteristic of this source. The molecular diagnostic ratios are the mutual proportions of the ambient concentrations of a single PAH or groups of PAHs that have similar physicochemical properties (Tobiszewski and Namieśnik 2012). Although the diagnostic ratios are more convenient in use than markers, they should be used cautiously because they are sensitive to atmospheric conditions and can be the same for different PAH sources (Dvorská et al. 2011).

In Table 3, some molecular diagnostic ratios (MDR) for the PAHs present in majority of the PM fractions in Katowice are presented. MDR computed for some PAHs in PM_0.03–0.26_, PM_0.26–1.6_, and PM_1.6–40_ indicate biomass and fossil fuel combustion as the PAH sources (Tobiszewski and Namieśnik 2012). The An/(An + Ph) and Ph/(Ph + An) suggest that a greater part of PM_0.03–0.26_ and the PAHs in it came from road traffic and that PM_0.26–1.6_ came from combustion of solid fuels. The Fl/(Fl + Py) indicates combustion of liquid fossil fuels as the source of the PM_0.26–1.6_-bound PAHs and combustion of coal, grass, and wood as the source of PM_0.03–0.26_- and PM_1.6–40_-bound PAHs (Ravindra et al. 2008; Tobiszewski and Namieśnik 2012).

**Table 3:** PAH molecular diagnostic ratios (MDR) for PM_0.03–0.26_, PM_0.26–1.6_, and PM_1.6–40_

MDR	PM_0.03–0.26_	PM_0.26–1.6_	PM_1.6–40_
An/(An + Ph)	0.67	0.30	–
Ph/(Ph + An)	0.33	0.70	–
Fl/(Fl + Py)	0.68	0.44	0.67
BaA/(BaA + Ch)	0.61	0.51	0.90
BaA/BaP	1.54	1.29	1.08
IP/(IP + BghiP)	–	0.33	–
BaP/BghiP	–	4.70	–

Each sample taking lasted several days, and the occurrence of artifacts, as well negative (evaporation of semi-volatile PAHs) as positive (adsorbing some gaseous PAHs on particles of EC, salts, mineral particles, etc.), during sampling cannot be excluded (Ravindra et al. 2008; Dvorská et al. 2011; Tobiszewski and Namieśnik 2012). Although the application of the cascade impactor prevented the drawn atmospheric aerosol from blowing through the filters, limiting the effects of these artifacts, and although for each PM basic fraction, the samples from the whole sampling period were combined into one sample to be analyzed for PAHs, still, the method used was not sensitive enough to determine the most stable PAHs, IP, and BghiP, in some very fine and coarse fractions of PM (Table 1), what makes the reasoning using MDR uncertain.

BaP, a well-studied five-ring hydrocarbon, is of special importance to environmental toxicology. It is one of the most mutagenic and carcinogenic hydrocarbons known (Nikolao et al. 1984; Ravindra et al. 2008). Its importance consists also in its being a basis for defining the toxic equivalence factor (TEF) and the carcinogenic equivalent (CEQ) for other PAHs. Namely, TEF for a PAH is defined relative to the TEF of BaP, the latter being assumed to be 1; CEQ of a group of PAHs is the linear combination of the TEFs and the ambient concentrations of these PAHs. TEF expresses the absolute toxicity of a particular PAH, CEQ—the toxicity of a group of ambient PAHs (Nisbet and LaGoy 1992). The mutagenic equivalent (MEQ) or the TCDD toxic equivalent (TEQ), defined in Rogula-Kozłowska et al. (2013b), can also be useful in assessing the influence of a PAH mixture on human health.

**Fig. 6:**
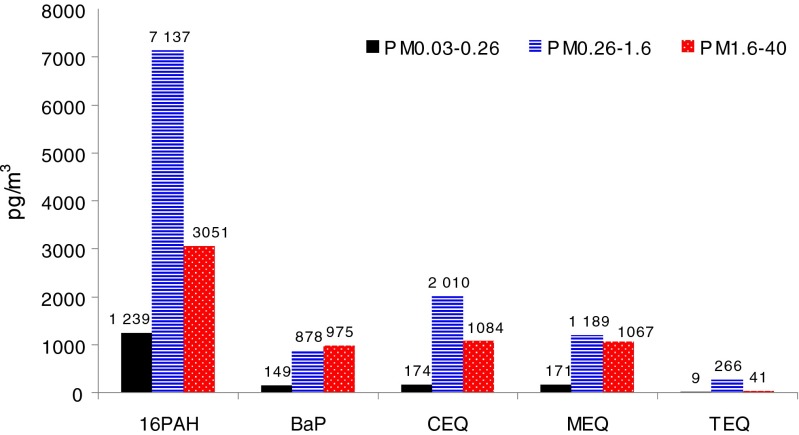
Concentrations of ΣPAH and BaP and values of CEQ, MEQ, and TEQ for PM_0.03–0.26_, PM_0.26–1.6_, and PM_1.6–40_

The ambient concentrations of ΣPAH and BaP from PM_0.03–0.26_, PM_0.26–1.6_, and PM_1.6–40_ and CEQ, MEQ, and TEQ for these fractions at the site in Katowice are presented in Fig. 6. Although PM_0.26–1.6_- and PM_1.6–40_-bound BaP concentrations are almost equal (concentration of PM_1.6–40_-bound BaP is a little higher than that of PM_0.26–1.6_-bound BaP), the PM_0.26–1.6_-bound ΣPAHs pose much greater risk to human health than the ΣPAHs from PM_1.6–40_. Although the mass share of ΣPAH in PM_0.03–0.26_ is greater than in PM_1.6–40_ (Fig. 5), the ambient concentrations of PM_1.6–40_-bound BaP and ΣPAH are very high, higher than those of PM_0.03–0.26_-bound BaP and ΣPAH. In Katowice, the health risk from coarse PM can be greater than the risk from very fine PM (Fig. 6).

## Conclusions

In Katowice, the partition of the particle (aerodynamic) diameters into three intervals, 0.03–0.26, 0.26–1.6, and 1.6–40 μm, by the stochastic behavior of the properties of ambient particles is very clear, and the fractions PM_0.03–0.26_, PM_0.26–1.6_, and PM_1.6–40_ obviously differ. Despite some technical limitations of the whole experiment, such as using in the computations the concentrations of PM components that were sampled at different times, duration of particular sample takings (several days), and arbitral simplifications in the chemical mass closure scheme, the revealed differences in the physicochemical properties of these three fractions are so systematic that the partition cannot be accidental but rather related with the origin of PM at the site (structure of emissions).

The average ambient concentration of PM_0.26–1.6_ was 14.5 μg/m^3^. By mass, PM_0.26–1.6_ was about 59 % of the total PM, PM_1.6–40_ was 28 %, and PM_0.03–0.26_ was 13 %. PM_0.03–0.26_ and PM_1.6–40_ consisted mainly of primary matter (64 and 59 % of their masses), and PM_0.26–1.6_ consisted of secondary matter (57 % of its mass). Thus, arising of the greater part of PM in Southern Poland depends on the conditions upon which the physicochemical transformations of primary matter in the air depend. Lowering of PM concentrations should therefore consist in imposing limits on emissions of precursory gases from combustion. Combustion is the primary source of PAHs, and, as it is shown in this paper, from among PM_0.03–0.26_, PM_0.26–1.6_, and PM_1.6–40_, the greatest health hazard from the PAH content in Katowice is posed by PM_0.26–1.6_.

The finest particles, mainly primary, coming usually from nucleation, condensation, or sublimation of organic (PAHs) or inorganic (oxides and salts) gases from combustion of coal, biomass, and liquid fuels, in Katowice are in PM_0.03–0.26_. Limits on the emissions from combustion, besides affecting the secondary part of PM_0.26–1.6_, will lower amounts of primary PM. It will perhaps not decrease essentially the PM (mass) concentrations, but it will significantly lower the health hazard from PM.

The coarsest particles, PM_1.6–40_, are mainly not only primary particles from erosion of road surface, constructions, cars, soil, etc., but also big agglomerates of soot and big particles of salts from combustion of solid fuels in household ovens or in the open air (burning plant material in allotment gardens, grass fires, etc.). They contain significant amounts of PAHs, and the health hazard from these PM_1.6–40_-bound PAHs can be higher than the health hazard from the PAHs contained in PM_0.03–0.26_.
